# *BlinkBoard*: Guiding and monitoring circuit assembly for synchronous and remote physical computing education

**DOI:** 10.1016/j.ohx.2024.e00511

**Published:** 2024-01-23

**Authors:** Andrea Bianchi, Kongpyung (Justin) Moon, Artem Dementyev, Seungwoo Je

**Affiliations:** a Department of Industrial Design, KAIST, Daejeon, Republic of Korea; b School of Computing, KAIST, Daejeon, Republic of Korea; c Google, Mountain View, CA, United States of America; d School of Design, SUSTech, Shenzhen, Guangdong, China

**Keywords:** Human–computer interaction, Embedded systems, Physical computing, Breadboard, Synchronous remote education

## Abstract

Motivated by the necessity of guiding and monitoring students when assembling electronic circuits during in-class activities, we propose *BlinkBoard*, an augmented breadboard that enhances synchronous and remote physical computing classes. *BlinkBoard* uses LEDs placed on each row of a breadboard to guide, via four distinct blinking patterns, how to place and connect components and wires. It also uses a set of Input/Output pins to sense voltage levels or to generate voltage output at user-specified rows. Our hardware uses an open protocol of JSON commands and responses that can be used directly via a command line interface to control the hardware. Alternatively, these commands can be issued within a front-end graphical application hosted on a computer for a more user-friendly interface, and to ensure bidirectional and real-time communication between the instructor’s guiding and monitoring panel, and all the students’ remote *BlinkBoards*. The *BlinkBoard* hardware is affordable and simple, partially due to a customized circuit configured via a hardware description language that handles the LEDs’ patterns with minimal load on the Arduino microcontroller. Finally, we briefly show *BlinkBoard* in use during a workshop with high-school students and an undergraduate design course.

## Graphical abstract

**Figure:**
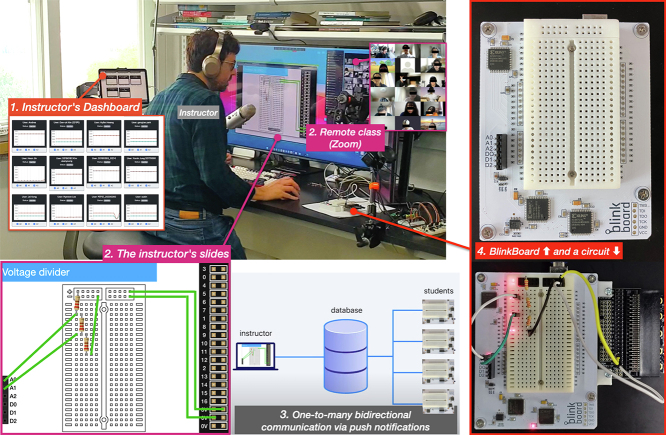

## Specifications table

**Table:** 

**Hardware name**	BlinkBoard

**Subject area**	Educational tools and open source alternatives to existing infrastructure

**Hardware type**	
	• Electrical engineering and computer science
	• Prototyping toolkit for education

**Closest commercial analog**	No commercial analog is available. Related research is highlighted in the text.

**Open source license**	MIT License

**Cost of hardware**	64 USD (rounded up)

**Source file live repository**	*Hardware files, HDL, firmware*: https://doi.org/10.5281/zenodo.6824753 or https://github.com/makinteractlab/BlinkBoard *GUI application (optional control software)*: https://github.com/makinteractlab/BlinkBoardApp

## 1 Hardware in context

It is well known that the most effective education is conveyed when students learn by making, rather than passively listening to information as bystanders or mere receivers [1]. This is particularly true for education that facilitates tinkering with electronics — a subject referred to as *physical computing* or *interaction prototyping* [2]. With physical computing, students of various backgrounds are encouraged to learn via projects by constructing circuits, often on a breadboard, with electronic parts, sensors, and actuators, whose behavior is typically programmed with microcontrollers such as the Arduino,^1^ Raspeberry Pi,^2^ or BBC micro:bit^3^ boards.

Hands-on activities have contributed to the popularity and growth of the physical prototyping toolkits, outreaching makers of various age groups (including children) and of various non-engineering backgrounds (e.g., design, art, etc...). However, at the same time, the necessity of physical proximity that enables these activities has also hindered the outreach to communities that are remote or have no access to traditional face-to-face education. In fact, while it is relatively easy to supervise the students’ hands-on progress for face-to-face classes and labs [3], developing online courses where experimentation plays an important role and that can support an indeterminate number of users from around the world (i.e., MOOC — Massive Open Online Courses) is significantly challenging [4].

Specifically, there are two types of challenges. The first challenge is that the instructor needs to be able to **guide the students** about how a circuit should be assembled. This was achieved in previous work by visualizing step-by-step instructions on how to connect electronic components using Augmented Reality [5], [6], [7], Virtual Reality [8], breadboards instrumented with LEDs [5], [9], and videos [10], [11] or graphical interfaces displayed on a computer [10], [12]. The second challenge is to enable an instructor to **monitor the students’ progress** and to check whether the students completed an exercise or encountered problems. Since individual monitoring rarely scales up for large classes, previous systems focused on the automatic detection of circuit problems with various types of interfaces [10], [13], [14]. The VISIR (Virtual Instrument Systems in Reality) remote lab [11] is a particularly interesting example that is deployed in dozens of universities [15], which supports monitoring of students’ progress during exercises with electronics. At its core the VISIR is an instrumented breadboard that allows wiring and measuring of electronic circuits remotely on a virtual workbench that replicates a physical circuit, bridging between remote digital schematics and the physical instantiation of a circuit on a server.

Motivated by these challenges and by prior work, we see an opportunity to integrate solutions that provide both **in-situ guidance of circuit assembly** and **monitoring of students’ progress** to quickly identify breakdown problems [13]. We present *BlinkBoard*, an open-source hardware platform that allows an instructor to remotely guide students on how to assemble a circuit step-by-step, using blinking patterns of LEDs to visualize connections directly on a breadboard (similarly to [5]). Furthermore, *BlinkBoard* enables a two-way implicit communication channel between the instructor and each of the students’ remote breadboard, by sampling digital and analog values from the circuit and sending them back in real-time to the instructor’s dashboard for supervision (Fig. 1.1). The key difference with previous work [5], [10] is that *BlinkBoard* supports a real-time bidirectional link between instructor and remote students, offering at the same time both guidance and monitoring. Because the prototype is meant for in-class education, the hardware is low-cost (≈64US$), easy to fabricate, and both hardware and software are open-source. Furthermore, compared to systems like VISIR, which share a similar goal, we take a different approach. While with VISIR the students interact with a digital representation of the circuit schematics using a software tool, and the circuit is physically “wired” remotely, in our approach the students interact directly with the hardware, while the instructions of how to assemble a circuit (the underlying schematics) are digitally dispatched from a remote server. In this paper, we mainly focus on describing the *BlinkBoard* hardware that supports this interaction, but we also explain how to operate the (optional) controlling application for in-class usage.

**Fig. 1:**
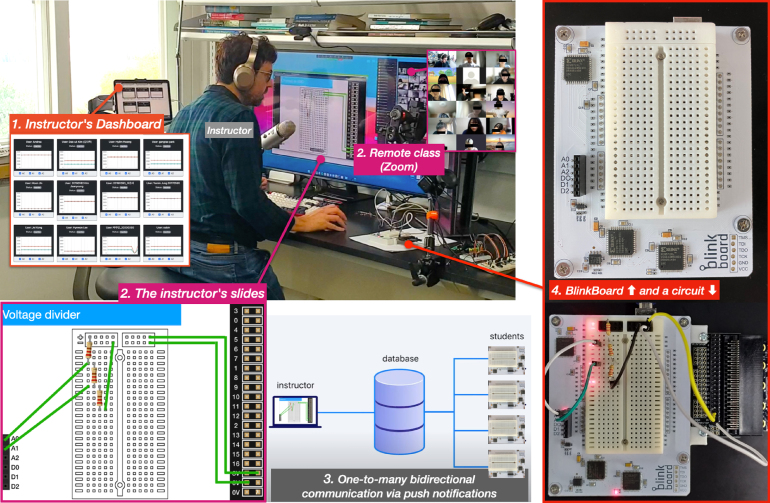
*BlinkBoard* is a physical computing prototyping toolkit that allows an instructor to guide (via visual patterns shown in hardware) and monitor in real-time how remote students construct circuits in a virtual classroom. ① An instructor can monitor voltage levels at specific points of all students’ circuits, or for a specific student ②. ③ The instructor uses slides showing step-to-step schematics instructions on how to place and connect the components of a circuit, while the *BlinkBoard* hardware visualizes these instructions in real-time directly on the breadboard via LED patterns.

**Fig. 2:**
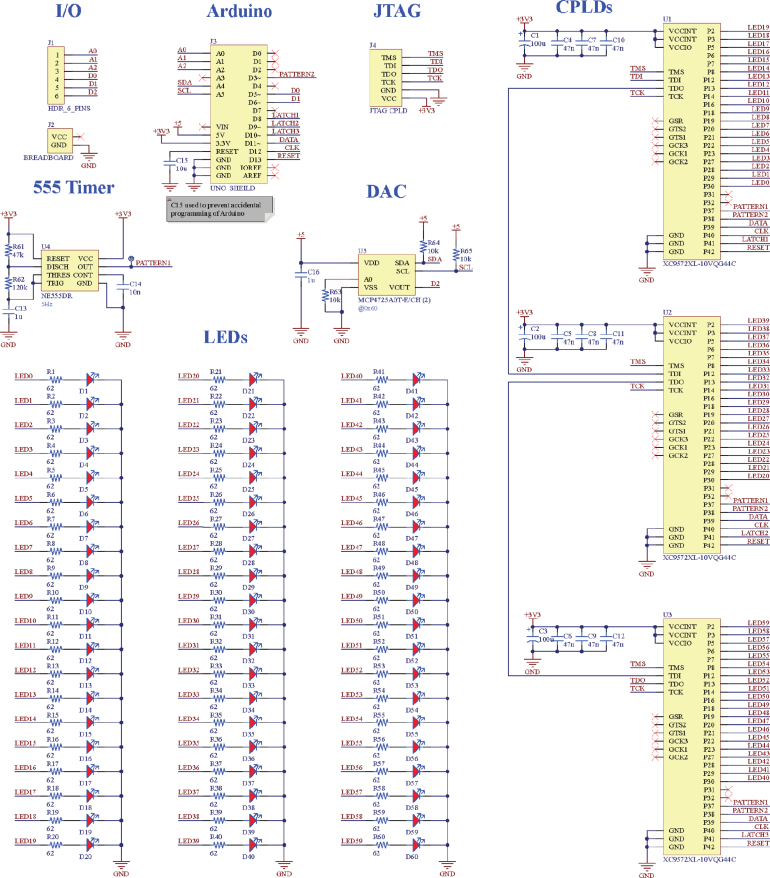
*BlinkBoard* electronic schematics.

## 2 Hardware description

*BlinkBoard* is a custom shield for the Arduino UNO,^4^ housing a custom printed breadboard (25 rows × 2 columns) and 50 LEDs placed on the side of each row as a visual indicator used for guiding the user about how to place components or wires. These LED indicators can be individually turned *on* or *off* or set to blink at two different frequencies (*slow* and *fast* pattern).

As shown in Fig. 2, the LEDs are driven by three integrated circuits (Xilinx XC9572XL CPLDs — Complex Programmable Logic Devices) configured with custom logic that, similarly to shift registers (e.g. 74HC595), can be cascaded and controlled in real-time via a latched serial-in interface. The circuits of the LED controllers running on the CPLDs were custom-designed and configured using the *Verilog* Hardware Description Language (HDL). In our configuration, the LEDs can be turned *on*, *off*, or set to *blink* in a continuous loop at two predetermined frequencies (2.5 Hz or 5 Hz) directly in hardware and without keeping the microcontroller occupied. In practice, this means that the Arduino is used only to configure the behavior of the hardware when needed and in real-time, while all CPU cycles are left for listening to incoming commands sent over USB serial. The behavior of these controllers is further described in the next subsection.

We chose to use CPLDs with custom logic rather than discrete off-the-shelf components (e.g., shift-registers like in prior work [5]) to minimize the number of components to be placed on the PCB (easier to design, cheaper to assemble), and to reduce the cost of parts and passive components (e.g., only require bypass capacitors). The Xilinx XC9572XL CPLDs, with their 72 macrocells and 1600 usable gates, allow developers to experiment with different features without having to re-design new boards to accommodate for changes and updates. We iteratively built the logic of the circuits based on basic building blocks (i.e., Verilog modules) to implement the shift register and the blinking of the LEDs (e.g., using clock divisions). We chose these CPLDs rather than more powerful FPGAs for simplicity (e.g., requiring fewer supporting parts), and because the XC9572XL chips offer built-in 5 V tolerant I/O pins that can be easily interfaced to the Arduino UNO (operating at 5V). A caveat of using CPLDs is that they are “older” technologies than FPGA, requiring to use legacy developing tools such as the Xilinx ISE Design Suite^5^ instead of the most recent Vivado Design Suite.^6^

The rest of the circuit (see Fig. 2 for an overview of the schematics) contains a 555 timer chip (U4) to generate the 5 Hz basic blinking pattern (58% duty-cycle), and a DAC – Digital to Analog Converter (U5) – for analog output. Six pins are exposed on the left side of the *BlinkBoard* to allow users to sense analog input (three analog pins of the Arduino) and three output pins (two digital pins connected to the Arduino and one analog pin connected to the DAC). As for the breadboard rows, each of the six pins has an LED indicator on the side. The shared ground between the *BlinkBoard* and the user’s circuit is provided via the ground (GND) bus of the breadboard (power buses are placed on the top of the breadboard, each with an LED indicator next to a *+* and *-* symbol). Finally, six JTAG pins are exposed for programming the CPLDs.

*BlinkBoard* implements two types of safety mechanisms to protect the software firmware and the hardware from students’ unintentional mistakes. Students might accidentally upload a different firmware on the Arduino that controls the *BlinkBoard* shield instead of another target device connected via USB. To avoid this situation, a capacitor (C15) is placed on the RESET pin of the Arduino, which effectively prevents a user from inadvertently overriding the firmware. To protect from erroneous wiring that might cause excessive current, *BlinkBoard* leverages the built-in protection mechanisms of the underlying Arduino board. Specifically, the Arduino UNO mounts a surface-mounted resettable fuse (Bourns MF-MSMF050-2) for overcurrent protection (500 mA), which prevents damage in case of accidental short circuits at any of the *BlinkBoard*’s exposed pins. In this sense, *BlinkBoard* achieves piggyback fault-tolerance on the Arduino’s design, without additional costs to the hardware. Although this is clearly a compromise and end-users can still damage the *BlinkBoard* hardware, we consciously opted for this design which trades off some security for simplicity and economic viability (e.g., ideally, a broken Arduino or hardware can be replaced at a low cost).

### 2.1 LED controllers design

**Fig. 3:**
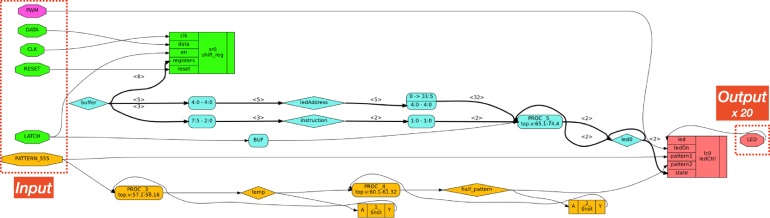
Simplified representation of the logic blocks described in the HDL on each of the three CPLDs.

Our custom LED controllers are 8-bit Serial-In, Parallel-Out Shift Registers devices, each capable of independently controlling 20 LEDs (the total number of LEDs is 60: 50 LEDs for the rows, 2 for the power busses, 6 for the I/O pins, and two 2 status indicators). Fig. 3 shows a simplified diagram of the logic blocks of our design, where, for clarity, we only show a single LED output (instead of 60 output LEDs). The code and the graph with the complete design can be found in the online repository.

Each controller takes a serial input (DATA, CLK) and a LATCH pin, like a typical shift register. The RESET pin allows resetting the hardware. The input pins are shown highlighted in  in Fig. 3. The shifted-in 8-input data (highlighted in  ) is interpreted as a 5-bit address for the LEDs and a 3-bit op-code to select one of the four patterns: *on*, *off*, *slow* (2.5 Hz), and *fast* (5 Hz). The third unused bit is for future extensions. The fast blinking pattern is supplied directly from the 555 timer chip (PATTERN_555 net), while the slower pattern is generated using a clock divider within the CPLD (in  ). Depending on the LED and pattern selected, the output LED (in  ) is set to be the input PWM signal from Arduino (in  ) modulated onto the selected blinking pattern. The reason for using a PWM input signal is that the brightness of all the LEDs can be controlled with a single signal.

**Fig. 4:**
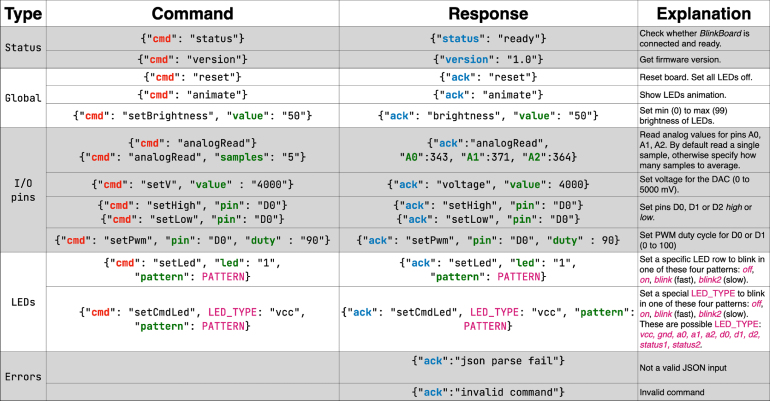
List of commands and responses in JSON format handled by the firmware.

### 2.2 Firmware operation and communication protocol

The firmware is flashed on the enclosed Arduino UNO (ATmega328P). A capacitor (*C15*) prevents users from inadvertently overriding the Arduino firmware (i.e., users can override the firmware only by opening the case and detaching the Arduino UNO from the shield). The firmware code depends only on two external open-source libraries: the ArduinoJson^7^ library for communication with the hosting PC, and the Adafruit MCP4725^8^ library for controlling the DAC.

The firmware operates by expecting input commands from a hosting computer connected via serial communication over USB at 115200 baud. All commands and responses are specified in JSON format and the full list of commands is shown in Fig. 4. Commands like **status** and **version** are used to query the current status of the hardware and the firmware version. Commands **reset**, **setBrightness** and **animate** turn *off* all the LEDs, specify globally their brightness level, or create an animation effect that lights up all the rows (i.e., the animation is used when the system is initially powered up).

**Fig. 5:**
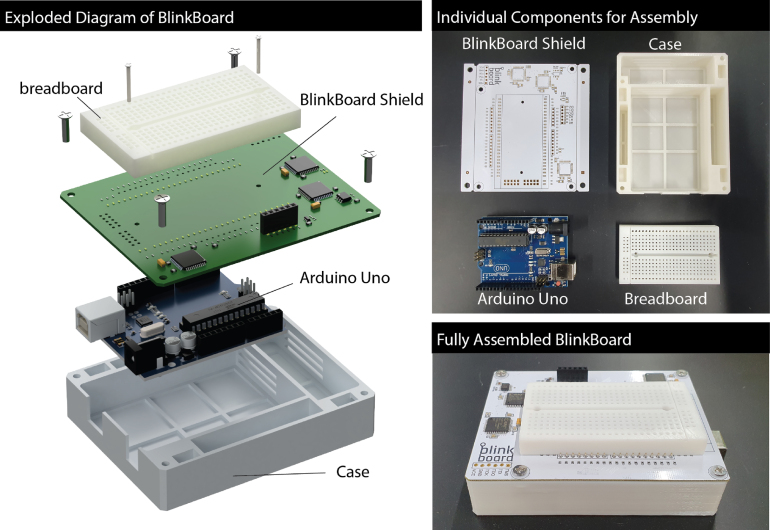
3D rendered models: the breadboard, the BlinkBoard shield, the Arduino UNO, and the outer case.

The six digital and analog pins on the side of the breadboard can be controlled with commands such as **analogRead** (read A0, A1 and A2 in millivolts, with optional number of samples), **setV** (set voltage in millivolts on D2), **setHigh**, **setLow** (on DO, D1 or D2) and **setPwm** (on D1, D2, with specified duty-cycle). Any LED indicators at the side of each breadboard row can be set to one of the four possible states (*off*, *on*, *blinking slow and fast*) with **setLed**. The LEDs placed next to the I/0 pins or next to the power busses (＋ and −) or the status LEDs can also be similarly controlled using the command **setCmdLed**. The firmware ensures that the command is correct and in valid JSON format, otherwise, an error would occur (*“json parse fail”* or *“invalid command”*) and the status LED indicating an error would turn on. The full list of commands accompanied by practical usage examples is available on the online repositories.

### 2.3 Hardware main keypoints

This is a short list of the unique aspects of the *BlinkBoard* hardware and how it could be used by other researchers.

• Arduino UNO compatible shield with 60 independently controllable LEDs, each capable of multiple blinking patterns built in hardware.
• The LEDs are controlled in hardware using programmable logic gates. This allows to microcontroller to focus on other tasks (e.g., sensing user input). A similar approach could be used in future work to control large numbers of actuators other than LEDs.
• The firmware uses an open protocol based on JSON messages for commands (*cmd*) and responses (*ack*).
• Hardware design is a result of cost-features and simplicity-complexity trade-offs. The resulting hardware costs 63.58 USD.

## 3 Design files summary

Fig. 5 shows an exploded diagram of all the *BlinkBoard*’s components. All 3D models were designed using Autodesk Fusion 360.^9^ The table below contains both the *CAD files* and the *3D printable* files in *stl* format. The *electronics* schematics files and the Printed Circuit Board (PCB) for the *BlinkBoard shield* were designed with Altium.^10^ We include in the online repository the source files, the PDF file with the schematics and assembly sheets, as well as the ready-to-fabricate *gerber* files. The *firmware* for the Arduino UNO was written in C++ using the PlatformIO^11^ developing environment. A reference to the source code can be found in the table below. Finally, the hardware description for the circuits on the CPLD devices was written in separate modules using Verilog. The files were synthesized with the Xilinx ISE Design Suite and uploaded to the CPLDs via the JTAG interface. All files in Table 1 are available on the project’s Github Repository found at https://github.com/makinteractlab/BlinkBoard. Files/folder names and paths are indicated relative to the root folder of the repository.

**Table 1:** Summary of design files.

Design filename	File type	License	Relative location of the file
BlinkBoard.SchDoc LEDcontroller.SchDoc LEDS.SchDoc	Schematics	MIT	/Electronics_Altium

BlinkBoard.PrjPcb	Layout	MIT	/Electronics_Altium

BlinkBoard.SchLib BlinkBoard.PcbLib	Components Library	MIT	/Electronics_Altium

Gerber.zip	Gerber	MIT	/Electronics_Altium/Output

Breadboard_fusion.f3d Case_fusion.f3d	CAD	MIT	/3D_models

Breadboard.stl Case.stl	3D model	MIT	/3D_models

main.cpp	Firmware	MIT	/ArduinoFirmware/src

constants.hpp	Firmware	MIT	/ArduinoFirmware/include

top.v ledCtrl.v shift_reg.v constraints.ucf top.jed	HDL/Verilog UCF/JED	MIT	/HDL_LedController/src

## 4 Bill of materials

The electronic components required to build the *BlinkBoard* are listed in Table 2, including their cost and possible suppliers. Prices may vary with the availability of components. The table below contains the parts of the *BlinkBoard* shield, with descriptors names as indicated in the schematics. Assembling the prototype also requires general parts and basic equipment like M1.6 and M3 nuts and bolts, soldering equipment, and tweezers.

**Table 2:** Summary of bill of material.

Designator	Component	Number	Cost per unit - USD	Total cost - USD	DigiKey part	Material type
C1, C2, C3	100 μF	3	0.73	2.19	T491A107 M004AT	Tantalum

C4 — C12	47 nF	9	0.17	1.53	CC0805JRX 7R9BB473	Ceramic

C13, C16	1 μF	2	0.14	0.28	CC0805ZRY 5V8BB105	Ceramic

C14	10 nF	1	0.10	0.10	CC0402KRX 7R6BB103	Ceramic

C15	10 μF	1	0.21	0.21	CC0805KRX 5R5BB106	Ceramic

D1 — D60	LED (red)	60	0.32	19.32	VLMS1500-GS08	

R1 — R60	62 Ω	60	0.02	0.96	RC0402FR-0762RL	Carbon Film

R61	47 kΩ	1	0.10	0.10	RC0402JR-0747KL	Carbon Film

R62	120 kΩ	1	0.10	0.10	RC0402JR-07120KL	Carbon Film

R63, R64, R65	10 kΩ	3	0.10	0.30	RC0402FR-0710KP	Carbon Film

U1, U2, U3	Xilinx CPLD 72 Macrocell	3	3.71	11.13	XC9572XL-10VQG44C	

U4	555 Timer	1	0.46	0.46	NE555DR	

U5	DAC 12-bit	1	1.28	1.28	MCP4725A0T-E/CH	

J1	Pin Header Socket 1×6 Straight (2.54 mm)	1	0.52	0.52	PPTC061LFBN-RC	

J3	Arduino UNO	1	23	23	1050-1024-ND	

J5,J8	Pin Header Single 1×8 Straight (2.54 mm)	2	0.4	0.8	61300811121	

J6	Pin Header Single 1×6 Straight (2.54 mm)	1	0.35	0.35	61300611121	

J7	Pin Header Single 1×10 Straight (2.54 mm)	1	0.95	0.95	61301011121	

Total				63.58		

## 5 Build instructions

**Fig. 6:**
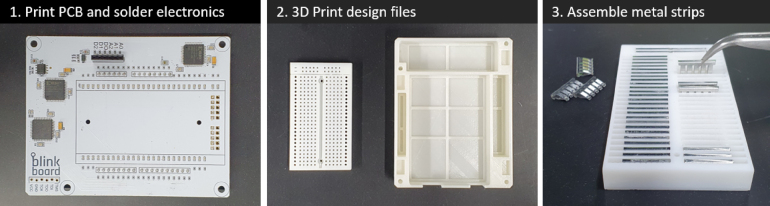
Steps 1-3 of the assembling process.

*BlinkBoard* can be reproduced by printing the design files (Table 1) and assembling the components listed in Table 2. The Fig. 6, Fig. 8 show the step-by-step guide to rebuild the *BlinkBoard*.

**Fig. 7:**
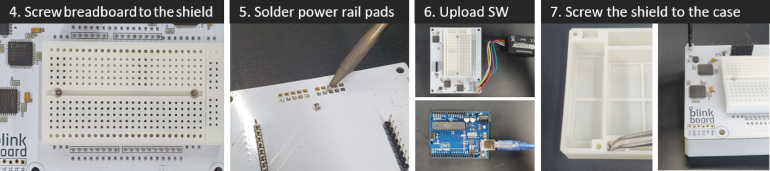
Steps 4-7 of the assembling process.

**Fig. 8:**
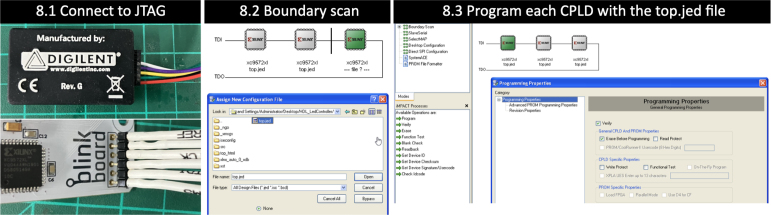
Steps for uploading the HDL on the CPLDs.

• **Step 1** The first step is to print the *BlinkBoard* PCB shield using the supplied *gerber* files and manually solder/assemble electronic components onto the printed shield. If placing an order from a manufacturer with a pick-and-place machine, provide them with the assembly instructions and the BOM file (see Fig. 7).
• **Step 2** 3D print the breadboard and the case using the *stl* files. For better resolution/precision of the pinholes of the breadboard, it is recommended to print the model with ABS-like white resin (Accura Xtreme White 200) via an SLA (Stereolithography) 3D printer. However, the bottom case can be printed simply with PLA (PolyLactic Acid) using a Fused Deposition Modeling (FDM) machine, ensuring low cost and strength.
• **Step 3** Assemble the metal clips placed underneath the breadboard to connect 2 four-hole female sockets on a row. These metal clips can be obtained by manually disassembling a 400-hole breadboard (e.g., Breadboard DM323).

• **Step 4** Place the breadboard on the shield and use two M1.6 8 mm bolts and nuts to tighten them.
• **Step 5** Solder the ground rail on the bottom to share all the grounds on the *BlinkBoard* shield. Make sure the ground socket of the breadboard is connected to this rail.
• **Step 6** Before connecting the shield to an Arduino, upload the firmware via USB.
• **Step 7** Insert the M3 nuts inside the housing slots of the case and fix the shield on the top of the 3D printed case using the M3 12 mm bolts.
• **Step 8** Generate the bitstream file from the hardware-description specifications (i.e. Verilog files) using the Xilinx ISE Design Suite and upload it via JTAG on each of the CPLDs. This step can be broken down into the following sub-steps: (8.1) Power up the assembled BlinkBoard hardware by plugging it into a computer via USB. Connect BlinkBoard to the XUP USB-JTAG Programming Cable^12^ as shown in Fig. 8. (8.2) On a PC opens the application **iMPACT** by Xilinx. This should automatically prompt for a boundary scanning of the connected devices, and ask to choose a file to upload to the CPLDs. Simply pick the *top.jed* file in the repository, or use the ISE Design Suite to regenerate a bitstream from the source Verilog files (HDL_LedController folder). For the success of this step, ensure that the JTAG is properly connected and that the BlinkBoard is powered up. (8.3) The icons of three CPLDs connected in series should be visible on the GUI. Select each one of them and click the “Program” button on the left panel to upload the bitstream on the CPLDs.

**Fig. 9:**
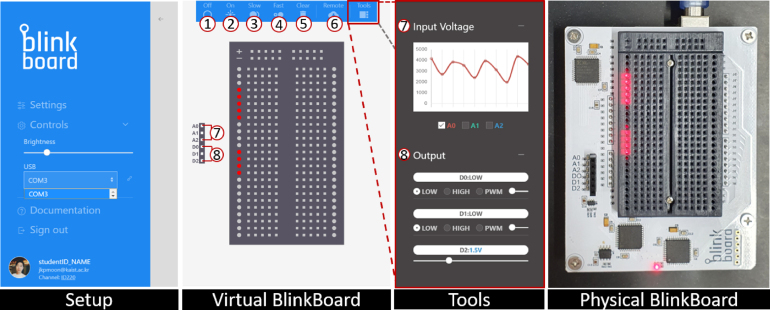
The different parts of the *BlinkBoard* GUI app. These include a side panel for configuration and setup, the Virtual Breadboard, the blinking patterns (1-4), and buttons for resetting the LEDs (5) and listening to remote commands (6). The *Tools* sidebar is used to inspect the input voltages (7) and control the output pins (8). The physical *BlinkBoard* on the right reflects the Virtual *BlinkBoard* in real-time.

## 6 Operation instructions

*BlinkBoard* can be operated in **two different ways**. The simplest way is using serial communication via a **command line interface**. After connecting the hardware to a hosting computer with a USB cable and establishing a serial connection at 115200 baud, the user can type the command *{“cmd”: “status”}\n\r* and expect to receive a *{“ack”: “ready”}* response (*\n\r* is the necessary command delimiter). Any command shown in Fig. 4 can be subsequently issued. While end-users might find direct control of *BlinkBoard* via the command line verbose and error-prone, this interface is suitable for debugging or for exposing hardware control to third-party applications. The user can issue the special command **help** (or alternatively the equivalent JSON command) directly from the command line to visualize a list of all the supported input commands and their descriptions.

The second method of operation is using a **graphical user interface (GUI)** that leverages, under the hood, the aforementioned serial communication and commands. We provide an example of this method with the open-source *BlinkBoard* app^13^ (Fig. 9). This front-end application enables end-users (students or instructors) to interact with *BlinkBoard*. Once a user is logged-in using a personal account, and after an initial one-time setup (e.g., selecting a USB port, setting the LEDs brightness level), the application connects to the *BlinkBoard* hardware, and the software displays a virtual breadboard. The user can then select blinking patterns by clicking buttons from the top menu, and assign blinking patterns at the desired rows by clicking on the corresponding rows of the virtual breadboard. These selections are immediately reflected in hardware. Furthermore, the user can inspect the voltage levels for the input pins A0, A1, and A2 on a graph in the *Tools* sidebar (Fig. 9.7) and use controllers to set voltage output values for pins D0, D1, and D2 (see Fig. 9.8).

However, the most interesting feature of the *BlinkBoard* app is that it allows an instructor to *guide* remote students, by sharing over the Internet a configuration of blinking patterns, causing all the remote connected *BlinkBoard* (i.e., the *Remote* feature in Fig. 9.6 is active) to instantly update and reflect this configuration. Effectively the instructor can share, step-by-step, the sequence of instructions to assemble a circuit, while, at the same time, remotely inspecting the voltage values for each of the students’ *BlinkBoards* via an online dashboard (Fig. 9.7). The software application can be further extended by displaying slides that visually show the assembly instructions step-by-step, where, at each slide, the remote *BlinkBoards* are updated.

The *BlinkBoard* app described above is a cross-platform application implemented in JavaScript using the Electron^14^ framework. The back-end manages the user authentication and the database which describes each of the users’ blinking configurations and I/O pins. The backend was developed using the Firebase^15^ suite by Google. The application and the source code are available online, but, being this paper focused on a hardware contribution, the full description of the software is beyond the scope of the paper.

**Fig. 10:**
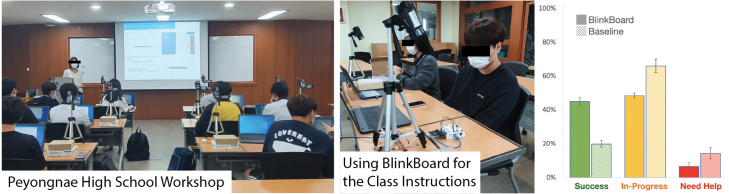
*BlinkBoard* was used in a workshop with high-school students (left and middle). Students’ activity logs comparison between group A and B (right).

## 7 Validation and characterization

To validate the efficacy of *BlinkBoard*, we ran a three-day physical computing workshop (Aug 17-20, 2021) with 21 high-school students at Pyeonae High School, in the city of Namyang, Republic of Korea (Fig. 10). The workshop was conducted *face-to-face*, and it was structured to test two separate conditions (*A/B testing*), wherein (A) 11 students used *BlinkBoard* and (B) 10 students used instead a conventional breadboard. The workshop was composed of basic electronics lectures with hands-on exercises and a hackathon project for the final day. During the exercises we used a custom-developed web app running on the students’ phones to track at a glance the students’ progress. Specifically, the app displayed three buttons indicating whether they finished the exercise or felt they were managing successfully their progress (green button), whether they were still working on the exercise (yellow button), or whether had difficulties and requested support from a teaching assistant (red button). We asked students to independently update their status while performing the exercises and recorded their input events on a database as a JSON file for subsequent analysis. To reduce noise in the data, consecutive user inputs were considered only if at least 5 s apart.

In general, the student and the instructor in the *BlinkBoard* group revealed that, in contrast with the baseline condition, they were able to focus more on the theoretical side of the content (how the circuit/sensors/actuators work) rather than on the wiring details of the circuits. These findings seem to be supported by our analysis of the activity logs—success, in progress, need help (see Fig. 10-right). A Two-Way ANOVA reveals statistical differences across the type of activities (F(2, 57)= 178.9, p≤0.01) and the interaction between activity and conditions (F(2, 57)= 40.6, p≤0.01). A post-hoc analysis with Bonferroni correction (alpha = 5%) reveals differences between each pair of log activities type. Furthermore, a comparison of the activity logs factor between the modality condition (*BlinkBoard* vs. baseline) shows differences for the number of success logs (p≤0.01), in-progress logs (p≤0.01), and need-help logs (p≤0.05). To explain these results in simple terms, it is visible from the graph in Fig. 10-right that students with *BlinkBoard* were much more confident achieving successful completion of the exercises, and requested less intervention from the TAs than not in the baseline condition. The data collected from this study are still being analyzed and a full analysis and report of the results is beyond the scope of this paper, but the data suggest good potential for applying *BlinkBoard* to synchronous face-to-face entry-level physical computing classes.

After the workshop, we also collected qualitative feedback about *BlinkBoard* from interviews with students and the high school teachers who monitored the workshop. All stakeholders expressed that *BlinkBoard* was useful in shifting the focus of learning toward the theoretical aspects of the lecture content (how the circuit/sensors/actuators work) rather than on the wiring details of the circuits shown in the slides. For example, the instructors noted that the two groups of students asked very different types of questions during the in-class activities. While the students in the baseline group asked more questions about how to connect components, in the *BlinkBoard* group students tended to ask questions more related to the general concepts and theory of electronics. Finally, the high school teacher who supervised the workshop described, during the interview, similar challenges when teaching students about electronics, stating they also spent considerable time helping them with the wiring of circuits, and remarking that “using *BlinkBoard* would make teaching this [how to wire circuits] very easy”. Overall, the interview results suggest good potential for applying BlinkBoard to synchronous face-to-face entry-level physical computing classes.

Finally, *BlinkBoard* was also tested by one of the authors as a teaching aid for the *online (remote)* course ID220 Interaction Prototyping offered by the Department of Industrial Design at KAIST, Korea. The ID220 course, a second-year elective class, was conducted during Fall 2020 and 2021 with 16 and 17 students respectively. It was advertised as an online course, which relied mainly on the Zoom^16^ video conferencing software, and in which *BlinkBoard* was used during online synchronous in-class activities. For example, the instructor would ask the students to create a voltage divider, showing step-by-step instruction slides synchronized with *BlinkBoard* about how to assemble the circuit, and how to wire different parts of the circuit to the platform’s I/0 pins. Simultaneously the instructor would be able to inspect, using an online dashboard, if students correctly completed the circuit by inferring from their voltage graph whether the components were connected as expected. This process is illustrated in Fig. 1. Like the workshop conducted in the high school, a full analysis and reporting of the results from these experiments are beyond the scope of the paper. However, the collected data anecdotally demonstrate that completely online and remote physical computing education can be aided by using *BlinkBoard* for guiding and monitoring students’ progress. We also think that *BlinkBoard* could have the potential for self-practice of exercises at home, although we currently do not have any data to support this claim, and future work will be necessary to see the effects of using *BlinkBoard* as a self-teaching aid.

## Ethics statements

All human participants involved in our workshops signed an informed consent about collection of data, and, in case of minors, the authorization was provided by their parents.

## CRediT authorship contribution statement

**Andrea Bianchi:** Funding acquisition, Investigation, Software, Writing – original draft, Writing – review & editing, Conceptualization, Data curation. **Kongpyung (Justin) Moon:** Data curation, Formal analysis, Writing – original draft. **Artem Dementyev:** Methodology, Resources, Writing – original draft, Writing – review & editing. **Seungwoo Je:** Conceptualization, Data curation, Supervision, Writing – original draft, Writing – review & editing.

## Declaration of competing interest

The authors declare the following financial interests/personal relationships which may be considered as potential competing interests: Andrea Bianchi reports financial support was provided by National Research Foundation of Korea (NRF).

## Appendix A Supplementary data

Video S1 Introduction video of BlinkBoard.

## Footnotes

12 https://digilent.com/shop/xup-usb-jtag-programming-cable/.

## Footnotes

1 https://www.arduino.cc.

2 https://www.raspberrypi.org.

3 https://www.microbit.org.

4 https://docs.arduino.cc/hardware/uno-rev3.

5 https://www.xilinx.com/products/design-tools/ise-design-suite.html.

6 https://www.xilinx.com/products/design-tools/vivado.html.

7 https://arduinojson.org.

8 https://github.com/adafruit/Adafruit_MCP4725.

9 https://www.autodesk.com/products/fusion-360.

10 https://www.altium.com.

11 https://platformio.org.

13 Project homepage: https://blinkboard.kaist.ac.kr.

14 https://www.electronjs.org.

15 https://firebase.google.com.

16 https://zoom.us.
